# Prospects for Using Expression Patterns of Paramyxovirus Receptors as Biomarkers for Oncolytic Virotherapy

**DOI:** 10.3390/cancers12123659

**Published:** 2020-12-05

**Authors:** Olga V. Matveeva, Svetlana A. Shabalina

**Affiliations:** 1 Sendai Viralytics LLC, 23 Nylander Way, Acton, MA 01720, USA; 2 National Center for Biotechnology Information, National Library of Medicine, National Institutes of Health, Bethesda, MD 20894, USA

**Keywords:** oncolytic viruses, oncolytic virotherapy, viral oncolysis, measles virus, Sendai virus, biomarkers, virus receptors, receptor retargeting, virus receptor expression, protein receptors, glycosphingolipid receptors, gangliosides

## Abstract

**Simple Summary:**

Some non-pathogenic viruses that do not cause serious illness in humans can efficiently target and kill cancer cells and may be considered candidates for cancer treatment with virotherapy. However, many cancer cells are protected from viruses. An important goal of personalized cancer treatment is to identify viruses that can kill a certain type of cancer cells. To this end, researchers investigate expression patterns of cell entry receptors, which viruses use to bind to and enter host cells. We summarized and analyzed the receptor expression patterns of two paramyxoviruses: The non-pathogenic measles and the Sendai viruses. The receptors for these viruses are different and can be proteins or lipids with attached carbohydrates. This review discusses the prospects for using these paramyxovirus receptors as biomarkers for successful personalized virotherapy for certain types of cancer.

**Abstract:**

The effectiveness of oncolytic virotherapy in cancer treatment depends on several factors, including successful virus delivery to the tumor, ability of the virus to enter the target malignant cell, virus replication, and the release of progeny virions from infected cells. The multi-stage process is influenced by the efficiency with which the virus enters host cells via specific receptors. This review describes natural and artificial receptors for two oncolytic paramyxoviruses, nonpathogenic measles, and Sendai viruses. Cell entry receptors are proteins for measles virus (MV) and sialylated glycans (sialylated glycoproteins or glycolipids/gangliosides) for Sendai virus (SeV). Accumulated published data reviewed here show different levels of expression of cell surface receptors for both viruses in different malignancies. Patients whose tumor cells have low or no expression of receptors for a specific oncolytic virus cannot be successfully treated with the virus. Recent published studies have revealed that an expression signature for immune genes is another important factor that determines the vulnerability of tumor cells to viral infection. In the future, a combination of expression signatures of immune and receptor genes could be used to find a set of oncolytic viruses that are more effective for specific malignancies.

## 1. Introduction

Oncolytic viruses are promising new agents for cancer treatment. They can kill cancer cells directly through infection or indirectly through activation of the immune system [1,2]. For the most effective virotherapy, elimination of malignant cells with a combination of both direct and indirect destruction is desirable. Like all viruses, oncolytic viruses use specific receptors to bind to and enter host cells. This review describes the tendency of tumor cells to overexpress certain viral receptors, but it also shows that, to varying degrees, these receptors are also expressed in many normal cells. However, regardless of whether cells are normal or malignant, absence of receptors for a particular virus makes the cells resistant to this virus infection. So, for better identification of individual patients who are most likely to benefit from virotherapy, their tumor cells should be screened for the presence of virus receptors. For many oncolytic viruses, such receptors are well characterized. Thus, simple tests that evaluate protein or RNA levels in tumor tissue could provide information about expression levels of a virus receptor.

Receptor mediated virus entry into a cell is only the first step in viral infection. Next, the virus must break through the cellular antiviral defense system, which usually effectively protects normal cells from any virus infection. Key players in such protection are interferons (IFNs); they help cells detect the presence of a virus and, in response, restrict proliferation, slow down metabolic processes, and trigger apoptosis [3,4]. However, malignant cells frequently have dysfunctional IFN pathways. Such dysfunction helps them to evade the immune system and survive, thus promoting tumor growth. The same IFN defects that help cancer cells escape immune surveillance make them vulnerable to virus infection [5]. Nevertheless, not all malignant cells have dysfunctional IFN pathways. Some of them can produce and/or respond to IFN signals and protect themselves from a virus infection. So, theoretically, even if a cancer cell had receptors for a particular oncolytic virus it still could be resistant to infection by the virus.

**Figure 1:**
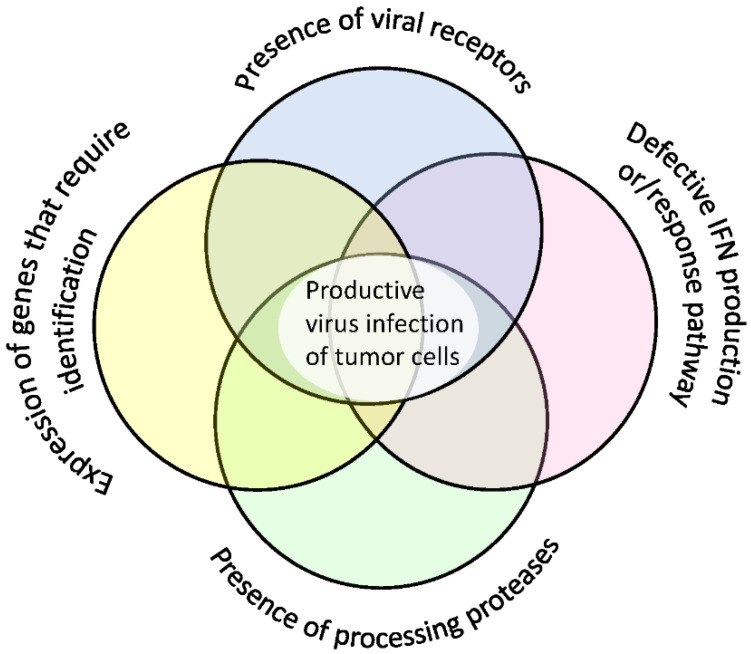
Factors influencing cells’ vulnerability to paramyxovirus infection. The host cell needs to (1) express virus receptors (2) have a malfunctioning IFN pathway, (3) express proteases responsible for proteolytic activation of virus fusion rotein, and (4) have other genes that require further identification.

Some viruses require cells to express processing enzymes that modify or cleave the viral proteins necessary for the formation of mature infectious virions. Thus, fusion protein in paramyxoviruses is synthesized as an inactive precursor and is activated through proteolytic cleavage by the cellular protease. Without such cleavage the virus is unable to sustain infection. For MV, this activating protease is furin [6] and for SeV it can be a number of serine proteases (TPSB2 [7,8,9], PRSS1 [10], PLG [11], F10 [12], and TMPRSS2 [13]). Some of these proteases are overexpressed in cancer cells [14,15,16]. In addition to those listed, the expression levels of other host genes influence vulnerability of cancer cells to a virus infection. Figure 1 illustrates factors necessary for a cell to become vulnerable to paramyxovirus infection.

To predict if a patient is likely to respond to oncolytic virotherapy, testing for the presence of virus receptors in tumor tissue is not sufficient. Additional tests are also needed to reveal the presence of impaired IFN signaling in the patient’s cancer cells, and the expression of virus processing enzymes and yet to be identified other proteins that accommodate virus infection. Currently, such tests are commercially unavailable and need to be developed to optimize patient selection protocols for future clinical trials.

**Figure 2:**
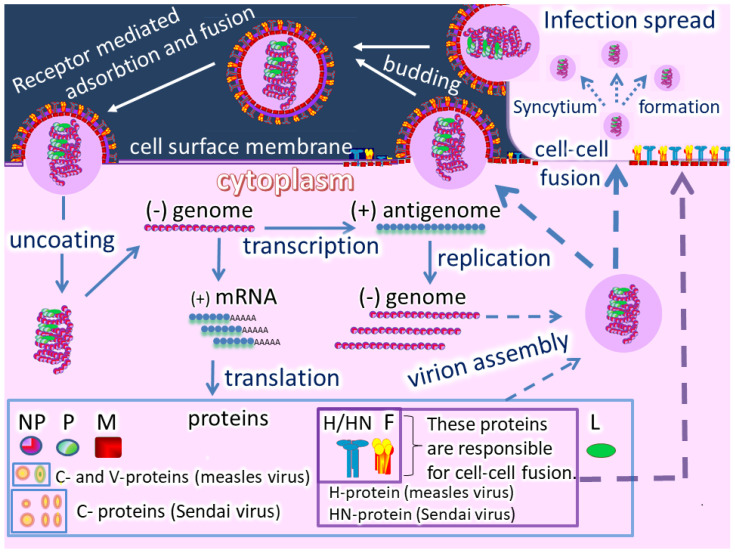
A visual representation of the cell cycle of measles virus (MV) and Sendai virus (SeV). Both viruses belong to the *Paramyxoviridae* family, but MV belongs to the *Morbillivirus* genus and SeV to the *Respirovirus* genus. The life cycles of MV and SeV are very similar, but there are several important differences. Their attachment to host cells occurs through different cell entry receptors and different viral cell attachment proteins. The MV virus uses an H protein with hemagglutinin activity, while SeV uses the HN protein with hemagglutinin (H) and neuraminidase (N) activities. In addition to these proteins, the genomes of these viruses encode 5 structural proteins and accessary proteins. The main structural proteins for both viruses are: Nucleoprotein (N), Phosphoprotein (P), Matrix protein (M), Fusion protein (F), and Large Protein (L). The MV genome encodes two non-structural proteins, C and V, [20], while the SeV genome encodes a set of non-structural proteins, collectively referred as C-proteins (C’, C, Y1, Y2, V, W) [21]. Viral replication for MV and SeV follows a negative-stranded RNA virus replication model in which genomic RNA (minus strand) is used as a template to create a copy of positive sense RNA, employing the RNA-dependent RNA polymerase embedded in the virion. The plus RNA is further used as a template for making multiple copies of the minus RNA. The plus RNA is also translated by the host’s ribosomes, producing all viral proteins. Viruses are then assembled from these proteins along with genomic RNA and budded from the host cell. Both MV and SeV can form syncytia by fusing neighboring infected and non-infected cells into a polykayion.

Oncolytic paramyxoviruses might become powerful anticancer agents [17,18,19]. Figure 2 shows the life cycle of the viruses, which can trigger syncytium (a polykarion) formation that protects virions from host neutralizing antibodies during intratumor virus replication and spreading.

So, two related processes can occur: Efficient intratumor virus spread and the resulting mass death of malignant cells. In general, oncolytic paramyxoviruses stimulate strong innate and adaptive anticancer immune responses by generating multiple danger signals. They are potent inducers of IFN and other immuno-stimulating cytokines, and they efficiently induce anticancer activity of natural killer cells, dendritic cells, and cytotoxic T lymphocytes [18]. Finally, the viruses require proteolytic cleavage of their fusion proteins by cellular serine proteases, which are sometimes overexpressed in cancer cells [14,15,16], and could add an additional level of specificity to viral oncolytic activity. Moreover, the gene that encodes a fusion protein in the paramyxovirus genome can be replaced with a constructed fusion protein that could be processed by tumor-associated matrix metalloproteases.

The purpose of this review is to summarize and analyze information related to expression patterns of receptors for oncolytic paramyxoviruses (both natural and artificially retargeted). In current literature and existing databases, receptor expression patterns are evaluated by quantitative and semi-quantitative measurements of RNA or protein. Analysis of the collected information may ultimately aid in the development of tests to identify oncolytic viruses that are more effective against specific malignancies. To compare expression levels in normal and cancerous tissues for each studied virus receptor, we analyzed a Human Protein Atlas (HPA) database [15,16,22]. HPA accumulates protein expression information from experiments performed by HPA project participants along with RNA-Seq information from The Cancer Genome Atlas (TCGA), Genotype-Tissue Expression (GTEx) and Functional Annotation of the Mammalian Genomes (FANTOM5) project databases [16]. We also analyzed relevant literature and gene expression patterns in the PubMed database.

Several paramyxovirus representatives have oncolytic properties. Among them are attenuated measles and mumps viruses, Newcastle disease virus, and SeV [17,18]. In this review, information related to MV and SeV receptors is compiled and analyzed.

## 2. Measles Virus as an Oncolytic Agent

**Figure 3:**
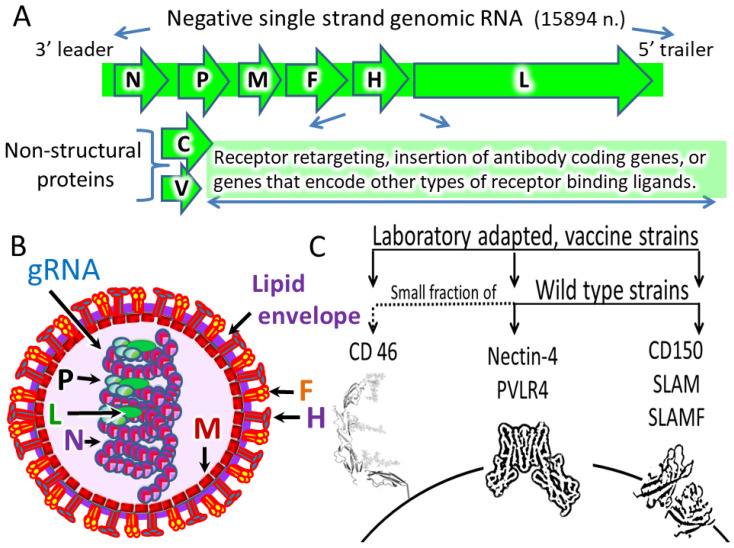
Schematic representation of the MV genome (**A**), virion (**B**) and host cell entry receptors (**C**). The RNA genome (gRNA) contains six transcription units that codes 6 main structural proteins: nucleoprotein (N), phosphoprotein (P), matrix protein (M), fusion protein (F), hemagglutinin (H), and large protein (L) RNA dependent RNA polymerase (RdRp). The viral genome also codes the nonstructural V and C proteins, which are antagonists of host innate immunity. The transcription units for each structural gene are separated by non-transcribed trinucleotide intergenic sequences and together are flanked by short leader and trailer sequences containing the genomic promoter (on the minus strand) and the antigenomic promoter (on the plus strand). Inside the virion, genomic RNA forms a complex with N, L, and P proteins. The virus is enveloped by a lipid membrane that has glycoproteins H and F associated with it as virion surface proteins. These proteins coordinate how the virus finds cells and enters them. For H protein, three receptors have been identified: Complement regulatory molecule CD46, the cell adhesion molecule nectin-4 and the signaling lymphocyte activation molecule (SLAM). MV wild-type strains use SLAM and nectin-4 as cell entry receptors. Vaccine strains and a small fraction of wild type strains, in addition, use CD46 as a cell entry receptor.

MV (Box 1, Figure 3) causes a highly contagious disease transmitted by respiratory aerosols that can trigger severe immunosuppression and even immune amnesia.

Box 1 **Measles Virus (MV).**

Taxonomy: The virus belongs to the genus Morbillivirus within the family Paramyxoviridae [23,24].
Host: Human
Origin: Most likely MV originated from a virus of non-human species.
Genome: MV has a single-stranded, negative-sense, non-segmented RNA genome that is ~16K nucleotides long.
Virion: MV is an enveloped virus with a lipid membrane.
Proteins: Nucleoprotein (N), phosphoprotein (P), matrix protein (M), fusion protein (F), hemagglutinin (H), large protein (L), and two nonstructural proteins C and V. Protein C is translated from the same mRNA as the P protein but using an alternative start codon in an overlapping ORF. Protein V is translated from an edited P mRNA.

Significant efforts by virologists in the second half of the 20th century were focused on finding a safe and effective vaccine against MV. In 1954, one MV isolated strain, when passaged in cell culture, gradually lost its pathogenicity, and became attenuated. From this attenuated variant of the virus, one of the first vaccine strain (Edmonston, denoted in the following text as MV-Edm) was obtained. Further passages of MV-Edm generated the more attenuated Schwarz and Moraten strains, which are still in use for vaccination against measles [24,25].

During the 20th century clinicians reported on isolated cases where measles disease relieved or caused remission of certain malignancies. Reports include descriptions of the regression of a number of hematological malignancies that took place after MV infection (reviewed in [26]). At the end of the century, virologists and oncologists started to investigate oncolytic properties of attenuated MV strains that are incapable of causing serious infection.

MV-Edm and its derivatives were the primary strains tested as oncolytic agents [27,28]. These strains can infect and kill a wide variety of cancer cells in vitro and in vivo. They are currently being investigated preclinically and in clinical trials for treatment of a large spectrum of malignancies, including ovarian, breast, head and neck cancers, as well as glioblastoma, multiple myeloma, mesothelioma, and T-cell lymphoma [27,28]. A virus with oncolytic properties can be delivered not only locally intratumorally, but also systemically, including by intraperitoneal and intravenous injection routes. In some trials patients survival compared favorably with that of historical controls and the side effects of the virotherapy were mainly mild [27,28].

## 3. Natural MV Receptors

For wild type and vaccine MV strains, the proteins CD150 (SLAM or SLAMF1) [29,30] and/or nectin-4 (also called poliovirus-receptor-like 4 (PVRL4)) [31,32,33] function mainly as cell entry receptors. A small fraction of wild type MV strains and all modern vaccine strains derived from the Edmonston strain also use CD46 receptors (Table 1, Figure 3C) [34,35].

RNA and protein expression patterns of MV receptors are estimated by several different approaches, including array technology, quantitative RT-PCR and RNA-Seq for RNAs, as well as immunohistochemical tissue staining, flow cytometry and western blotting for proteins. Table 1 and Table 2 respectively summarize expression patterns of natural MV receptors described in normal and malignant cells.

CD150 (also called signaling lymphocytic activation molecule 1 (SLAM or SLAMF1)) is a transmembrane glycoprotein member of the signaling lymphocytic activation molecule family. It modulates the activation and differentiation of a wide variety of immune cells and is involved in the regulation of both innate and adaptive immune responses [36,58,59,60]. CD150 is expressed on the surface of hematopoietic stem and progenitor cells including natural killer cells, dendritic cells, and memory B and T cells [36,37,38] (Table 1). Therefore, wild-type MV can infect various cells that are involved in the host’s immune response, resulting in strong immunosuppressive effects and erasure of immune memory from previous pathogen infections, causing immune amnesia [61,62].

In Japan, a set of MV-based oncolytic constructs was produced by removing MV’s ability to interact with CD150 [63,64,65]. The removal of interaction ability between a virus and its receptor is called “blinding”. It has been demonstrated that “blinding” results in the pathogenicity loss of the constructs, effectively making the MV strains non-infective to monkeys [66]. However, the constructs maintain the ability to kill malignant cells, including tumors, in model animals [63,64,65]. Three types of human malignancies were tested as murine xenografts, including breast, pancreatic, and lung carcinomas. The animals treated with intratumoral injections of the experimental constructs survived longer (Table 3).

Nevertheless, some malignancies do overexpress CD150. Many cell lines generated from Hodgkin’s and Burkitt’s lymphomas are characterized by very high levels of CD150 mRNA and proteins [51]. Both types of lymphomas can sometimes regress after natural infection with wild-type MV (reviewed in [26]). It is likely that these malignancies could be successfully targeted by an oncolytic virus that interacts with CD150 as a cell entry receptor. This hypothesis is supported by observations that MV-Edm can infect metastases and primary tumors in mantle cell lymphoma murine xenografts [67]. Perhaps other cancers that express high levels of CD150 could also be successfully targeted by attenuated MV with the ability to interact with CD150.

CD46 (complement regulatory protein or membrane cofactor protein) is a membrane glycoprotein that serves as a regulator of the complement system. By inhibiting complement activation in host cells, this protein protects cells from complement associated damage [68]. CD46 is also involved in other processes: It interacts with at least seven human pathogens and regulates the adaptive immune response by inducing differentiation of T cells into regulatory T cells [69,70].

According to HPA, CD46 protein is expressed at comparatively high levels in glandular cells of the breast, stomach, and colon as well as in several other cell types. At low levels it is expressed in almost all human cells and tissues [14,15,16]. CD46 expression protects a cell from complement-dependent cytotoxicity, so expression in a cancer cell promotes escape from immune surveillance and provides the cancer cell with a strong survival advantage [71]. Therefore, advanced cancers are frequently characterized by high levels of CD46 (Table 2). Two studies demonstrate that efficiency of cellular virus entry into and killing of tumor cells correlates with CD46 cell surface protein expression [52,72]. The cell vulnerability to virus infection and syncytia formation has been shown to correlate with the level of CD46 expression. Thus, at a low expression level of CD46, which is typical of normal cells, infection occurs, but intercellular fusion is negligible. However, tumor cells with a higher CD46 expression are much more vulnerable to virus infection along with the formation of syncytia [72].

Based on these observations, CD46 could be included in a list of biomarkers to predict potential tumor response to virotherapy by attenuated MV.

Nectin-4 (also called PVRL4—Poliovirus-Receptor-Like 4) is a transmembrane protein that belongs to a family of Ca^2+^-independent immunoglobulin-like cell adhesion molecules. It contributes to cell to cell adhesion and intercellular communication [73,74] and serves as a receptor for MV (Table 1, Figure 3A). According to HPA, the nectin-4 protein is expressed at high or medium levels in glandular cells of breast, colon, gall bladder, and stomach as well as in some other cells and tissues [14,15,16].

Nectin-4 gene expression promotes malignant cells’ evasion of growth constraints related to matrix detachment. This gene has been frequently found to be amplified and overexpressed in some solid tumors [75,76]. A summary of malignancies in which nectin-4 has high expression levels is presented in Table 2. Most likely, this protein helps MV-Edm to enter a malignant cell. The observation that nectin-4 was specifically used for MV entry into nectin-4 positive cancerous breast and colon cells supports this hypothesis [45]. Therefore, nectin-4 is another candidate to be included in a list of sensitivity biomarkers for oncolytic attenuated MV.

## 4. MV Retargeting for Binding New Cancer-Associated Proteins

Cellular entry of MV-Edm requires the interaction between viral H protein and cell surface receptors. In many types of normal cells, expression of CD150, CD46, or nectin-4 is detected. To make the oncolytic virus safer by targeting cancer cells more specifically, it would be beneficial to modify the virus preference for host cell entry. By changing viral H protein, which is responsible for the virus–receptor interaction, MV-Edm can be retargeted to infect different cells.

The affinity of the receptor for the virus is an important determinant of infectivity and, consequently, infection-induced cell fusion. In this context, affinity is a measure of the strength with which a virus binds to a cellular receptor. The fusion of infected cells with each other depends on this affinity and on the density of receptors on the cell surface, which is a measure of the concentration of receptors in the cell membrane. This density depends on the levels of intracellular expression of the receptor that can be measured. There is a threshold for receptor expression below which cell fusion is ineffective. There is also another threshold for receptor expression, above which there is no further increase in membrane fusion in cell culture [77,78]. Because of this non-linear relationship between viral infection-induced cell fusion and the level of expression of the receptor in vivo, it is very important to test any retargeting strategy.

Retargeting can be achieved by inserting genes that encode single-chain fragments of antibodies or other receptor-binding ligands into the viral genome (Figure 3A). This genetic engineering procedure allows for the creation of MV constructs that target a wide range of cancer associated proteins. Several studies describe such virus retargeting modifications (Table 4). The retargeting is a result of genomic changes that enable MV to use different cancer cell associated proteins as cell entry receptors (Table 4).

A proof of the possibility of redirection of MV cell targeting was obtained with engineered MV-Edm based constructs targeting three cancer associated proteins: EGF, IGF1 [79], and carcinoembryonic antigen (CEA) [80]. In cell cultures, MV-Edm constructs can successfully target, infect, and destroy corresponding malignant cells that overexpress one of these three proteins.

The retargeting genetic engineering approach was further used for creation of viral constructs with ability to recognize two other membrane associated proteins. One was phosphoprotein CD20 [81], which is expressed in normal hematopoietic cells and overexpressed in B-cell lymphomas, leukemias and some other malignancies (Table 5). Another was glycoprotein CD38 [82], expression of which characterizes immune cells and overexpression characterizes NK/T-cell lymphomas, lymphocytic leukemias, multiple myelomas and other malignancies (Table 5). Antitumor effects of both constructs were tested in animal xenografts. Implanted CD20-positive fibrosarcomas demonstrated delayed growth after IP construct delivery, while CD38-positive multiple myeloma cells became less tumorigenic after premixing with the corresponding viral construct.

An alternative approach for MV-Edm retargeting to cell surface antigens of choice was developed by using cystine knot proteins instead of single chain antibodies [83,111]. These short proteins are capable of binding integrins, which are frequently overexpressed by a tumor’s vascular endothelium. The retargeted virus was able to infect and kill cancer cells that expressed the integrins, including glioblastoma, medulloblastoma, melanoma and others [111]. Most importantly, when injected intravenously into animals carrying glioblastoma, the construct reached the tumor and caused cytopathic effects [111].

The introduction of the ability to bind new viral receptors may be accompanied by blinding to the natural receptors CD150 and CD46. This blinding reduces the potential infection of CD150- and CD46-positive normal cells because CD150 is expressed on the surface of normal hematopoietic stem and progenitor cells [36,37,38], while high CD46 expression characterizes glandular cells of many organs [14,15,16]. Blinding increases the safety of viral construct application because MV infection is usually detrimental to host healthy cells.

Blinding to specific receptors became possible after identification of residues in H protein that are necessary for CD150 or CD46 binding [112]. Multiple retargeting strategies have been used to modify the MV-Edm genome. These include the genomic introduction of genes encoding single-chain antibody fragments (scFv), cystine knot proteins, and designed ankyrin repeat proteins (DARPins). The antibody fragment strategy allowed the targeting of various cancer associated proteins such as CD38, epidermal growth factor receptor 1 [113,114], folate receptor 1 [109], prostate specific membrane antigen [110], human epidermal growth factor receptor 2 (HER2/neu) [77], prominin or CD133 [115,116], and plasminogen activator urokinase receptor [117,118]. The cystine knot strategy targeted integrins, which are highly expressed in glioblastomas, medulloblastomas and melanomas [111]. Finally, the DARPin strategy targeted ovarian carcinomas that express HER_2_/neu or/and epithelial cell adhesion molecule (EpCAM) [119,120], along with EGFR-expressing glioblastomas [121]. To reduce bystander killing of receptor-expressing normal cells, a gene that encodes an engineered viral fusion protein and that can be processed by tumor-associated matrix metalloproteases was added to the virus genome. This introduction made the targeting of the virus to tumor cells very specific [121]. All of these new constructs were tested in cell lines and some in xenograft models of human malignancies, and demonstrated strong or moderate antitumor effects (Table 6).

## 5. SeV as an Oncolytic Agent

SeV causes respiratory infections in mice and other rodents (Box 2). However, it is not associated with any human disease and can be a safe oncolytic agent. Safety of SeV for humans, including small children, has been confirmed experimentally. The virus in the form of nasal drops has been tested as a vaccine against human parainfluenza virus type 1 (HPIV-1), which causes respiratory symptoms in humans. Testing in adults and children demonstrated that experimental administration of wild type SeV triggered production of neutralizing antibodies towards HPIV-1 and was well tolerated [123,124]. Replication competent SeV vector showed an excellent safety profile in a stage 1 clinical trial [125] and is considered highly suitable as an antigen delivery tool [126,127]. Pre-existing anti-vector immunity didn’t affect the immunogenicity of SeV-delivered antigens [126].

Box 2 **Sendai Virus (SeV).**

Taxonomy: The virus belongs to the genus Respirovirus within the family Paramyxoviridae [23].
Host: The virus causes respiratory infections in mice, hamsters, guinea pigs, rats, and other rodents [128].
Genome: SeV has single-stranded, negative-sense, non-segmented RNA genome that is ~15K nucleotides long [129].
Virion: SeV is an enveloped virus with a lipid membrane.
Proteins: Nucleoprotein (N), phosphoprotein (P), matrix protein (M), fusion protein (F), hemagglutinin-neuraminidase (HN), large protein (L), and nonstructural proteins collectively referred as C-proteins (C’, C, Y1, Y2, V, W) that are translated from an alternative RNA transcript of the P gene [129].

Wild-type SeV can replicate and productively infect a large spectrum of malignant cells ex vivo (Table 7). A pilot study demonstrated that some canine mastocytomas can be eradicated with the help of SeV injections [130]. However, the virus is infectious and immunosuppressive for laboratory rodents. Therefore, for studying SeV oncolytic properties in a rodent model, a set of viral constructs that are nonpathogenic for experimental mice was created. These constructs were tested in animals bearing a variety of human xenograft tumors including sarcoma, melanoma, pancreas, colon, hepatocellular and prostate carcinomas. The SeV constructs promoted growth suppression or even complete tumor eradication of these malignancies [131,132,133,134], and elimination of established brain tumors [135].

In addition, UV- inactivated SeV virions have immune-stimulating properties: In syngeneic mice they promote immunomodulated tumor regression of colon [136,137], bladder [138], and kidney [139] cancers. In murine xenografts these virions contribute to the eradication of human prostate cancer [140].

So far, SeV has not been widely tested in clinical settings. An attempt to treat one case of human leukemia with a set of viruses, including SeV, was undertaken in 1964 at the Clinical Research Center of University Hospitals of Cleveland. Short-term remission in one patient affected by acute leukemia was observed after IV virus injection [153]. A few patients affected by various malignancies were treated with intradermally or intratumorally injected SeV in Moscow (Russia) in the 1990s. In a small fraction of patients treated with the virus, primary tumors and metastases disappeared, even when virotherapy was a monotherapy. These patients experienced a pronounced long-term remission that sometimes lasted more than 5–10 years [154].

## 6. SeV receptors

**Figure 4:**
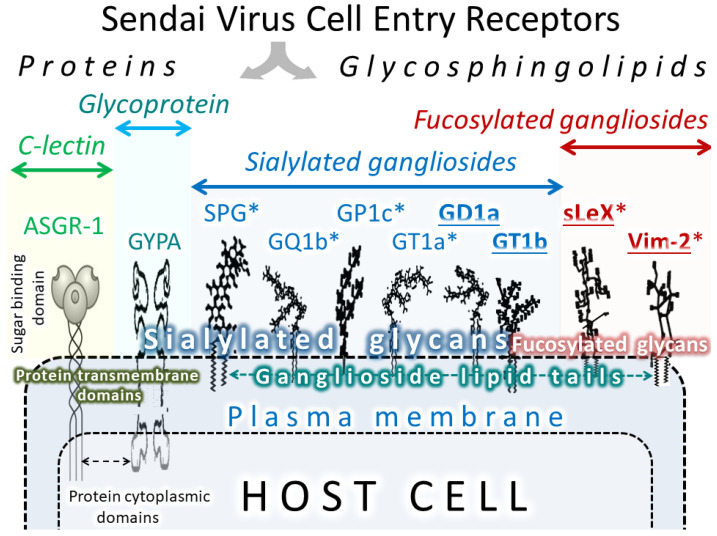
SeV receptors. The names of receptors with known high binding affinity to the virus are marked with stars. The names of receptors that are overexpressed in some malignancies are in bold and underlined.

SeV, as a representative of respiratory viruses, uses mainly molecules containing sialic acid residues (sialylated proteins as well as lipids) as cell entry receptors. Thus, bovine sialoglycoprotein (GP2) [155], human asialoglycoprotein receptor (ASGR1) [145,156], and sialoglycoprotein (glycophorin A (GYPA)) [157] bind SeV with high affinity and could act as virus receptors. Glycans (polysaccharides) attached to lipids could also bind SeV and serve as entry receptors. For example, two carbohydrates, sialyl Lewis-x and VIM-2, when attached to lipids, are capable of binding to SeV with high affinity [158]. Other SeV receptors are represented by gangliosides (Table 8, Figure 4).

Sialyl-Lewis X antigen (sLeX), also called stage-specific embryonic antigen 1 or cluster of differentiation 15 (CD15s), is one of the most important blood group antigens. It is a tetrasaccharide and may be attached to a lipid or a protein [92,93,94]. SLeX demonstrated high binding affinity to SeV when attached to sphingolipid [158]. However, whether sLeX can bind with a virus when attached to a protein is unknown. Expression of sLeX correlates significantly with malignant cell invasion, tumor recurrence and overall patient survival for an extremely broad range of cancers (Table 9) (reviewed in [167]). It is likely that sLeX positive cancer cells use the leukocyte adhesion pathway for extravasation, which facilitates tumor invasion and spread. Tumors with high expression of this antigen can bind SeV and are potential candidates for SeV therapy.

VIM-2 antigen, (also called cluster of differentiation 65 sialylated [CD65s]), is a carbohydrate that can be attached to a sphingolipid and has high binding affinity to SeV [158]. VIM-2 is expressed on surfaces of granulocytes, normal myeloid cells, and cells of acute myeloblastic leukemias [160,184]. Its expression is critically important for extravascular infiltration of acute myeloid leukemia cells [185]. Perhaps myeloblastic leukemias that are resistant to modern therapies could be treated with SeV.

Gangliosides are sialic acid-containing glycosphingolipids that are capable of binding SeV. It has been demonstrated that these molecules can serve as SeV cell entry receptors (Table 7). There is substantial evidence that at least three of them, SPG, GD1a, and GT1b, are highly involved in carcinogenesis and metastasis (Table 8). High expression of SPG characterizes lymphoid leukemia cells [189,190] and GD1a characterizes breast cancer stem cells [186]. High expression of both SPG and GD1a was found in castration-resistant prostate cancer cells [187]. High expression of GT1b is universally associated with brain metastases that originate from an extremely broad spectrum of cancers [188].

GM3, GD1a, and GT1b expression in a cell might be less predictive of SeV infectability than the expression of other molecules (104). Therefore, sLeX, VIM-2 and SPG are potential biomarkers for identification of cancers that could be efficiently infected by the virus. However, it is likely that new receptors for the SeV virus will be identified in the future.

Cellular expression of gangliosides is currently evaluated using glycan-specific antibody-based methods. These methods are not always suitable for large-scale screenings. Moreover, anti-ganglioside monoclonal antibodies are not always commercially available [170]. Therefore, indirect measurement of ganglioside expression through expression levels of fucosyltransferases and glycosyltransferases, which are enzymes that finalize ganglioside synthesis, represents an alternative. Expression of these enzymes and production of gangliosides are highly correlated [187]. At least five representatives of the fucosyltransferase family and six representatives of the glycosyltransferase family are responsible for synthesis of gangliosides that could serve as SeV receptors (Table S1). All these proteins are frequently overexpressed in various tumors and their expression levels correlate with tumor metastatic status and duration of patient survival (Table S2). These enzymes deserve to be studied as potential biomarkers of the oncolytic infectivity of SeV.

## 7. Potential Problems of Virus Delivery and Retargeting

### 7.1. Preexisting Immunity

One potential problem of MV and SeV applications as oncolytic agents is pre-existing antiviral immunity, which might affect systemic tumor-targeted viral delivery and intratumoral infection spread. For MV, this immunity is a result of childhood vaccination or measles disease. For SeV, previous infection with human parainfluenza virus type 1 (HPIV1) causes this immunity to the extent that the two viruses share some antigenic determinants [123,124]. It has been shown for SeV [191] and for MV [192] that prevalence of specific neutralizing antibodies against these viruses in adult human population is extremely variable. It is still largely unknown to what degree preexisting immunity may decrease the effect of oncolytic virotherapy. However, some virus delivery approaches discussed in the next section might help minimize this problem.

### 7.2. Virus Transportation and Tumor Delivery

Oncolytic virus constructs can be preloaded to specific cell carriers ex vivo and subsequently, after intravenous injection, transported to tumor sites in vivo [193,194]. Hypothetically, dendritic cells (DCs) could serve as oncolytic virus transportation vehicles. These cells can be infected by both vaccine and wild type strains of MV [195] via the CD150 cell receptor [196]. MV-infected DCs have higher motility toward the epithelial cell layer compared to uninfected ones. Therefore, MV infection enables rapid trafficking of the virus toward epithelial cells [197] and, perhaps, to other tissues including malignant tumors and metastases. Blinding of MV constructs to CD150 increases virus safety but might decrease efficiency of virus tumor delivery through its natural cell carriers such as DCs. SeV can also infect DCs [198] and can transform them into activated mature cells that efficiently contribute to tumor clearance and animal survival [199]. It is not known if MV-Edm or SeV-loaded DCs could ensure viral antibody protection and efficient tumor delivery; however, it is likely. Researchers should not ignore this hypothetical natural route of virus delivery to the tumor. For example, reovirus-loaded DCs protect the virus from neutralizing antibodies and facilitate viral infection of transplanted melanoma cells in model animals [200,201]. The ability of DCs to migrate after viral infection may facilitate viral tumor delivery without detection by host pre-existing immunity. Consequently, this ability might be a great asset for oncolytic virotherapy.

## 8. Additional Factors Determining Cell Sensitivity to Viruses

The presence of viral receptors is a necessary but not sufficient condition for a cell to be vulnerable to viral infection. Despite the presence of the receptors, a malignant cell can be resistant to the virus when the functioning of the IFN pathway is not impaired [202]. If the pathway is active in the cancer cell and the IFN signal is transduced from the cell surface to its nucleus, the cell can be protected from viral infection.

The cells’ ability to activate constitutively expressed genes of the IFN response pathway (ISGs) was the main prognostic factor for detection of carcinomas resistant to MV-Edm. Virus resistance of ovarian carcinomas and gliomas was linked to characteristic expression patterns of 22 ISGs [28]. Similar results were obtained for other malignancies; the sensitivity of melanoma cells to attenuated MV was associated with their response to type I IFN, even though MV receptor levels were the same among the tested cells [203].

The absence of certain proteases is another reason for the inability of the host cell to produce infectious virus. Expression of type II transmembrane serine proteases (TTSPs) by a cell is critical for the proteolytic activation of paramyxovirus F-proteins. Furin serves as an F-protein activating protease for MV [6] while other proteases such as PSB2 [7,8,9], PRSS1 [10], PLG [11], F10 [12], and TMPRSS2 [13]) serve a similar function for SeV. A host cell that does not express high enough levels of TTSPs can produce only noninfectious virions, rather than infectious virus particles. Expression patterns of TTSPs are variable among malignant cells and some cancers’ progression has been shown to relate to alteration of these patterns [204]. Therefore, in some cancer cells that express paramyxovirus F-protein activating TTSPs, the virus can undergo multiple rounds of infection, whereas in other cells which do not express these proteases, the virus can undergo only one, if any, rounds of infection.

In addition to those listed above, there are probably many more genes and proteins that affect the vulnerability of cancer cells to viral infection. Therefore, more research is needed to determine the target tumor cell pathways responsible for productive viral replication, post-replication processing, assembly, and budding of virions.

## 9. Conclusions

The cell entry receptors of oncolytic paramyxoviruses are represented by different types of molecules such as proteins and glycans. The molecules that serve as viral receptors for attenuated MV and SeV have extremely variable expression patterns in malignancies. A reliable predictive model for categorization of tumor cells according to their susceptibility to oncolytic virus infection requires many input parameters, which include but are not limited to expression patterns of viral receptors. Most likely, gene signatures of several immune-related genes such as IRGs and certain type II transmembrane serine proteases may serve as useful input parameters for predictive models. The creation and verification of a multi-parameter predictive model should have measurable therapeutic benefits for oncolytic virotherapy.

## Figures and Tables

**Table 1:** Natural receptors for MV and their expression in normal cells.

Receptor	High Expression in Normal Cells	Expression Evaluation
CD150/SLAM	Hematopoietic stem and progenitor cells including T, B, natural killer, and dendritic cells [36,37,38]	Multicolor flow-cytometry
	Spleen red pulp cells and thymus cortical and medullary cells [14,15,16]	Immunohistochemical tissue staining AB: HPA069319,CAB002438
Nectin-4/PVLR 4	Glandular cells of breast, stomach colon, gall bladder and others [14,15,16]	Immunohistochemical tissue staining AB: HPA016903,CAB010401
CD46/membrane cofactor protein	Glandular cells of breast, stomach, colon and others [14,15,16]	Immunohistochemical tissue staining AB: HPA010775

Abbreviations: AB: Antibodies.

**Table 2:** Expression of natural MV-Edm receptors in malignancies.

Malignancy	CD150/SLAM (Ref/Evidence)	CD46/Membrane Cofactor Protein (Ref/Evidence)	Nectin-4 (Ref/Evidence)
Breast cancer		[39]/IS, [14,15,16]/IS, TCGA dataset	[33,40,41,42,43,44,45]/IS, FC, PCR
Cervical cancer		[14,15,16]/IS, TCGA dataset	
Colorectal cancer		[14,15,16]/IS, TCGA dataset, [46]/oligo-array	[14,15,16,33,45,46]/IS, TCGA dataset
Endometrial cancer		[14,15,16]/IS, TCGA dataset	
Glioma		[47] IS, FC	
Liver cancer		[14,15,16]/IS, TCGA dataset	[48]/PCR, IS
Lung cancer			[33,49]/IS, ELISA
Non-small cell lung cancer		[50] IS, FC	[46]/Oligo-array
Lymphoma	[51]/PCR, IS, WB, FC	[52] IS, FC	
Melanoma		[14,15,16]/IS, TCGA	
Multiple myeloma		[53]/IS, [46]/Oligo-array	
Ovarian cancer		[54]/IS, WB	[55,56]/PCR, IS, WB, FC
Pancreatic cancer		[14,15,16]/IS, TCGA dataset	[57]/IS
Prostate cancer			
Stomach cancer			
Thyroid cancer			[14,15,16]/IS, TCGA dataset
Urothelial cancer		[14,15,16]/IS, TCGA dataset	

Abbreviations: IS: Immunohistochemical staining; FC: Flow Cytometry; PCR: Quantitative RT-PCR; TCGA: Tissue Cancer Genome Atlas; WB: Western immunoblotting.

**Table 3:** Retargeting MV receptors by blinding to CD150.

Blinding to	Effect	Cell Type or Malignancy	Model and Type of Virus Delivery to Animals	Reference
CD150/SLAM, no natural CD46	Viability of CD150-positive lymphoid cells unaffected; reduced infection of CD46-positive primary normal human cells; tumor stabilized or regressed	Breast carcinoma	Xenografts; IT virus delivery	[63]
	Tumor stabilized; animal survival prolonged	Pancreatic carcinomas	Xenografts; IT virus delivery	[64]
		Lung carcinoma		[65]

**Table 4:** Retargeting MV for binding to new receptors.

Blinding to	Introducing Property to Bind	Effect	Cell Type or Malignancy	Model and Type of Virus Delivery to Animals	Reference
	Via fusion of viral H protein with epidermal growth factor (EGF) or insulin-like growth factor 1 (IGF1) receptor binding domains				
None	EGF or IGF1 receptors	Infection of EGF or IGF1 receptor positive and CD46 negative cells	EGF or IGF1 receptor positive cells	Cell culture	[79]
	Via fusion of viral H protein with a single-chain variable fragment (scFv)				
	Carcino-embryonic antigen (CEA)	Infection of CEA positive cells	CEA-positive cells	Cell culture	[80]
	CD20	Delayed growth	Fibrosarcoma	Xenografts; IP virus delivery	[81]
	CD38	Malignant cells less tumorigenic, animal survival prolonged	Multiple myeloma	Xenografts; construct premixed with tumor cells before implantation	[82]
	Via fusion of viral H protein with echistatin, which is a 49-residue peptide from family of disintegrins				
	Integrin alpha(v)beta3	Tumor regressed or stabilized	Multiple myeloma	Xenografts; IT virus delivery	[83]

**Table 5:** Expression of retargeted measles virus receptors in normal and malignant cells.

Receptor Name	Alternative Name	Expression	
		In Malignancies	In Normal Cells and Tissues
CEA glyco-proteins	Carcinoembryonic antigen-related cell adhesion molecules	High or moderate in gastric, colorectal, lung ovarian, breast, and cervical cancers [84]	Different subfamily members expressed to different degrees in hematopoietic cells, glandular cells of colon, etc. [14,15,16]
CD133	Prominin-1 (PROM1)	High in leukemias [85], gliomas [86,87], colorectal, prostate, endometrial, pancreatic and thyroid cancers [14,15,16], and non-small cell lung cancers [88]. Levels in gliomas [87] and non-small cell lung cancers [88] are negatively correlated with patient survival [87]	High in glandular cells of gall bladder, endometrium, cervix and uterus [14,15,16]. Also expressed on the surfaces of hematopoietic stem cells [89], epithelial progenitor cells [90], and neural and glial stem cells [86]
CD20	MS4A1, B1, Bp35, CVID5, LEU-16, MS4A2, S7, membrane spanning 4-domains A1	Frequently high in B-cell lymphomas [91], B-cell leukemias [92], and melanoma stem cells [93]. Less frequent in Hodgkin’s lymphoma [94], myeloma [95], and thymoma [96]	Low and moderate expression in white and red pulp in spleen, hematopoietic cells of bone marrow, lymphoid tissues of appendix. and other tissues [14,15,16]
CD38	Cyclic ADP ribose hydrolase	High in chronic lymphocytic leukemia [97,98] in NK/T-cell lymphomas [99], and in multiple myeloma [100]	High in large spectrum of immune cells as well as glandular cells of prostate and seminal vesicles [14,15,16]
Epidermal growth factor receptor 1 (EGFR1)	ErbB 1, HER1	Particular high in gliomas, high in renal, urothelial, lung, liver, and many other cancers [14,15,16]	Low levels in a number of normal tissues but high levels in trophoblastic cells of placenta [14,15,16]
Epidermal growth factor receptor 2 (EGFR2)	Receptor tyrosine-protein kinase, ErbB-2, HER2/neu, ERBB2, CD340	Frequently highly overexpressed in malignancies including breast [101], stomach [102], endometrial [103,104], ovarian, uterine [105] colorectal [106], thyroid [107], urothelial [108]	Medium levels in glandular cells of appendix, breast, and cervix, myocytes, respiratory epithelium, and urothelial cells [14,15,16]
Insulin-like growth factor receptor (IGF1R)	IGF-1 receptor	High in lymphomas, thyroid, liver, pancreatic, and many other cancers [14,15,16]	Low level of expression in bone marrow hematopoietic cells, respiratory cells, and glandular cells of gallbladder [14,15,16]
Folate receptor 1 (FOLR1)	Folate receptor alpha, Glutamate carboxypeptidase II (GCPII), and folate hydrolase 1	High in ovarian cancers [14,15,16]; particularly strong and frequent expression of mRNA observed in non-mucinous ovarian cancers [109]	Medium levels in brain, lung, and salivary gland tissues [14,15,16]
Prostate specific membrane antigen, (PSMA)		High expression in malignant prostate cells [110]	High expression in prostate tissues [110]
Urokinase receptor	UPA, UPAR, CD87, PLAUR	Infrequently expressed in malignant cells [14,15,16]	High in bone marrow, lymphoid tissues, neutrophils, and respiratory epithelial cells of the nasopharynx and bronchus

**Table 6:** MV receptor blinding and retargeting.

Blinding to	Introducing Property to Bind	Effect	Cell Type or Malignancy	Model; Route of Virus Delivery to Animals	Reference
CD150/SLAM and CD46	Via fusion of viral H protein with scFv				
	CD38 or EGFR	Tumor stabilized; animal survival prolonged	CD38 or EGFR positive cancers	Xenografts; IT or IV	[113,114]
	Folate receptor 1 (FOLR1)	Biodistribution more specific towards malignant tissues; tumor stabilized; animal survival prolonged	Ovarian cancer	Xenografts; IV	[109]
	Prostate specific membrane antigen, (PSMA)	Tumor stabilized; animal survival prolonged	Prostate cancer	Xenografts; IT	[122]
	HER2 protein	Malignant cells infected in vitro, tumor regressed, animal survival prolonged	Ovarian cancer	Xenografts; IP	[77,78]
	CD133, Prominin1 (PROM1)	Tumor formation inhibited; animal survival prolonged	Glioblastoma, lung metastases of colon cancer and hepatocellular carcinoma	Xenografts; IT or IV	[115,116]
	Urokinase receptor	Delayed development of lung metastases, animal survival prolonged	Breast cancer	Syngeneic and xenografts; IV	[117,118]
	Via fusion of viral H protein with cystine knot proteins				
	Integrins	Malignant cells killed in vitro; cytopathic effects produced in vivo	Glioblastoma, medullo-blastoma, melanoma	Glioblastoma xenografts; IV	[111]
	Via fusion of viral H protein with designed ankyrin repeat proteins (DARPin)				
	Bispecific binding to HER2/neu, and/or EpCAM	Animal survival significantly prolonged, tumor burden reduced	Ovarian cancer	Xenografts; IT	[119,120]
	EGFR	Malignant cells killed in vitro	Glioblastoma multiforme	Cell lines	[121]

**Table 7:** Cancer cell-lines susceptible to SeV infection.

Cell Line	Type of Malignancy	Reference
Human origin		
MCF7	Breast carcinoma	[141]
HeLa	Cervical carcinoma	[142]
CaCo2	Colon carcinoma	[13]
U118	Glioblastoma	[143]
U87MG	Most likely, human glioma	[144]
Hep G2	Hepatic carcinoma	[142,145,146]
Huh7		[146,147]
A549	Lung carcinoma	[142,148,149,150]
Calu-3		[13]
U937	Histiocytic lymphoma	[149]
Namalwa	Burkitt’s lymphoma	[149,151]
PC-3	Prostate carcinoma derived from metastatic site in bone	[152]
DU145	Prostate carcinoma derived from metastatic site in brain	
Murine origin		
4T1	Mammary gland metastatic adenocarcinoma	[142]

**Table 8:** Cell entry receptors for sendai virus.

Sub-Type of Molecule	Receptor	Affinity to SeV	Ref.	Function in Normal Human Cells	Expression in Normal Human Cells
Glycoproteins					
Human asialoglyco-protein receptor 1	ASGR1	High	[145,156]	Removes the target glycoproteins from circulation in the liver	Hepatocytes [14,15,16]
Bovine glycoprotein 2	Glycoprotein2/GP2	High	[155]	-	
Human sialo-glycoprotein	Glycophorin A/GYPA/CD235a	High	[157]	Defines the antigenic determinants for some blood groups	Bone marrow, immune cells, [14,15,16]
Fucosylated glycans					
Tetra-saccharide	Sialyl Lewis-x antigen (sLeX/CD15s)	High	[158]	Serves as a blood group antigen and participates in cell-cell recognition process.	Bone marrow, erythrocytes [14,15,16]
Ceramide-dodeca-saccharide	VIM-2 antigen (CD65s)			Unknown	Granulocytes and monocytes [158]
Sialylated gangliosides					
Ganglio-series	GD1a, GT1b, and GQ1b,	Not reported	[159]	Cell-cell recognition, adhesion, and signal transduction	Granulocytes, normal myeloid cells [160]
	GT1a, GP1c	High	[161,162,163]	-	Many cell types, but mainly the cells of the nervous system [164]
	GD1a, GT1b	Moderate	[161,162,163]	-	
	GQ1b	Very high			
	GM3	Low	[165]	Cell–cell recognition	Blood cells, liver
Neolacto-series	Sialosylparagloboside (SPG, NeuAcα2-3PG)	Very high	[163,166]	-	Common for non-neural cells
	NeuAcα2-3I NeuAcα2-3i		[165]	-	
	NeuGca2-3I NeuAca2-6PG NeuAca2-6I	Moderate		-	

**Table 9:** Receptors for SeV and their expression in malignancies.

Receptor	Malignancy/Effect of Receptor Expression	Ref.	Monoclonal AB Availability
Human asialoglyco-protein receptor 1	High expression in liver cancer and occasionally moderate expression in gliomas, renal, pancreatic, colorectal, and ovarian cancers	[14,15,16]	Two variants [14,15,16]
Sialyl-Lewis^x^ Antigen (sLeX/CD15)	Non-small cell lung cancer/enhances post-operative recurrence	[168,169]	Many variants [170]
	Lung cancer, distant metastases	[171]	
	Colorectal cancer/promotes liver metastases, decreases time of disease-free survival	[172,173,174]	
	Gastric cancers/decreases patient survival time	[175,176]	
	Breast cancer/decreases patient survival time	[177,178,179]	
	Prostate tumor/promotes bone metastases	[180,181,182]	
	Cell lines of variable origin/high expression enhances adhesion of malignant cells to vascular endothelium	[183]	
	Variable cancers/high expression related to lymphatic invasion, venous invasion, T stage, N stage, M stage, tumor stage, recurrence, and overall patient survival	Review [167]	
VIM-2 antigen (CD65s)	Acute myeloblastic leukemias	[160,184,185]	One variant [170]
GD1a	Breast cancer stem cells	[186]	Many variants [170]
	Castration-resistant prostate cancer cells	[187]	
GT1b	Brain metastases from colon, renal, lung, esophagus, pancreas, and mammary carcinomas	[188]	Three variants [170]
SPG	Castration-resistant prostate cancer cells	[187]	One variant [189]
	Lymphoid leukemia cells	[189,190]	

## Supplementary Materials

The following are available online at https://www.mdpi.com/2072-6694/12/12/3659/s1, Table S1: Processing transferases for Sendai virus receptors; Table S2: Overexpression of processing transferases for Sendai virus receptors in malignancies.

## Abbreviations

CEA	Carcinoembryonic antigen
EGF	Epidermal growth factor
EGFR	Epidermal growth factor receptor
FOLR1	Folate receptor 1
HER2/neu	Tyrosine-protein kinase erbB-2 (human epidermal growth factor receptor 2)
IFN	Interferon
IP	Intraperitoneal delivery
IT	Intratumoral delivery
IV	Intravenous delivery
MV	Measles virus
PVRL4	Poliovirus-receptor-like 4, molecule (nectin-4)
SeV	Sendai virus
SLAM/SLAMF1/CD150	Signaling lymphocytic activation molecule 1
SPG	Sialosylparagloboside
