# Supplementary material for: Prospects for Using Expression Patterns of Paramyxovirus Receptors as Biomarkers for Oncolytic Virotherapy
Source: Cancers (Basel). 2020 Dec 5;12(12):3659. doi: 10.3390/cancers12123659 (PMC7762160; doi:10.3390/cancers12123659)
Supplement: Supplementary file 1 [file cancers-12-03659-s001.pdf]

Supplementary Materials:

Prospects for Using Expression Patterns of Paramyxovirus Receptors as Biomarkers for Oncolytic Virotherapy

Olga V. Matveeva and Svetlana A. Shabalina

Table S1. Processing transferases for Sendai virus receptors..

Receptor. Ganglioside (Sialilated Glycosphingolipid)	Glycosyltransferase	Type of Malignancy	Ref.
GD1a	ST3Gal1	Glioblastoma (ST3Gal1 defines metastatic invasiveness.)	[1]
		Ovarian carcinoma	[2]
		Breast carcinomas	[3]
		Colorectal cancer	[4,5]
		Bladder cancer (ST3Gal1 is high in cancers with tendency to recurrence.)	[6]
GD1a, GT1b	ST3GAL2	Colorectal cancer	[4]
		Breast cancer stem cells	[7]
Sialo-sylparagloboside (SPG)		One major ganglioside of lymphoid leukemia	[8,9]
SPG	ST3GAL6	Hepatocellular carcinoma	[10]
		Multiple myeloma (ST3GAL6 influences malignant cells homing and survival.)	[11]
GD1a	ST6GALNAC4	Lung cancer metastases	[12]
GD1a, GT1a and GQ1	ST6GALNAC5	Colorectal carcinomas (SNP polymorphism is related to occurrence of brain metastases)	[13]
		Breast cancer (ST6GALNAC5 enhances metastatic cells adhesion to brain endothelial cells and passage through the blood-brain barrier.)	[14]
GD1a	ST6GALNAC6	Lung adenocarcinoma	[15]
GT1a, GT1b		Renal cancers	[16]
GP1c and GQ1		Colon cancer	[17]

Table S2. Overexpression of processing transferases for Sendai virus receptors in malignancies.

Receptor Ganglioside (Sialilated Glycosphingolipid)	Glycosyltransferase	Type of Malignancy	Ref.
GD1a	ST3Gal1	Glioblastoma (ST3Gal1 defines metastatic invasiveness.)	[7]
		Ovarian carcinoma	[8]
		Breast carcinomas	[9]
		Colorectal cancer	[10,11]
		Bladder cancer (ST3Gal1 is high in cancers with tendency to recurrence.)	[12]
GD1a, GT1b	ST3GAL2	Colorectal cancer	[10]
		Breast cancer stem cells	[13]

Sialosylparagloboside (SPG)	ST3GAL6	One major ganglioside of lymphoid leukemia	[14,15]
SPG		Hepatocellular carcinoma	[16]
		Multiple myeloma (ST3GAL6 influences malignant cells homing and survival.)	[17]
		Melanoma	[18-20]
GD1a	ST6GALNAC4	Lung cancer metastases	[21]
GD1a, GT1a and GQ1	ST6GALNAC5	Colorectal carcinomas (SNP polymorphism is related to occurrence of brain metastases)	[22]
		Breast cancer (ST6GALNAC5 enhances metastatic cells adhesion to brain endothelial cells and passage through the blood-brain barrier.)	[23]
GD1a	ST6GALNAC6	Lung adenocarcinoma	[24]
GT1a, GT1b		Renal cancers	[25]
GP1c and GQ1		Colon cancer	[26]

References

1. Britten, C.J.; Bird, M.I. Chemical modification of an alpha 3-fucosyltransferase; definition of amino acid residues essential for enzyme activity. *Biochim. Biophys. Acta* **1997**, *1334*, 57–64.
2. Ngamukote, S.; Yanagisawa, M.; Ariga, T.; Ando, S.; Yu, R.K. Developmental changes of glycosphingolipids and expression of glycogenes in mouse brains. *J. Neurochem.* **2007**, *103*, 2327–2341, doi:10.1111/j.1471-4159.2007.04910.x.
3. Okajima, T.; Chen, H.H.; Ito, H.; Kiso, M.; Tai, T.; Furukawa, K.; Urano, T.; Furukawa, K. Molecular cloning and expression of mouse GD1alpha/GT1aalpha/GQ1balpha synthase (ST6GalNAc VI) gene. *J. Biol. Chem.* **2000**, *275*, 6717–6723.
4. Chandrasekaran, E.V.; Xue, J.; Xia, J.; Locke, R.D.; Patil, S.A.; Neelamegham, S.; Matta, K.L. Mammalian sialyltransferase ST3Gal-II: its exchange sialylation catalytic properties allow labeling of sialyl residues in mucin-type sialylated glycoproteins and specific gangliosides. *Biochemistry* **2011**, *50*, 9475–9487, doi:10.1021/bi200301w.
5. Hatano, K.; Miyamoto, Y.; Nonomura, N.; Kaneda, Y. Expression of gangliosides, GD1a, and sialyl paragloboside is regulated by NF-kappaB-dependent transcriptional control of alpha2,3-sialyltransferase I, II, and VI in human castration-resistant prostate cancer cells. *Int. J. Cancer* **2011**, *129*, 1838–1847, doi:10.1002/ijc.25860.
6. Vandermeersch, S.; Vanbeselaere, J.; Delannoy, C.P.; Drolez, A.; Mysiorek, C.; Guerardel, Y.; Delannoy, P.; Julien, S. Accumulation of GD1alpha Ganglioside in MDA-MB-231 Breast Cancer Cells Expressing ST6GalNAc V. *Molecules* **2015**, *20*, 6913–6924, doi:10.3390/molecules20046913.
7. Chong, Y.K.; Sandanaraj, E.; Koh, L.W.; Thangaveloo, M.; Tan, M.S.; Koh, G.R.; Toh, T.B.; Lim, G.G.; Holbrook, J.D.; Kon, O.L., et al. ST3GAL1-Associated Transcriptomic Program in Glioblastoma Tumor Growth, Invasion, and Prognosis. *J. Natl. Cancer Inst.* **2016**, *108*, doi:10.1093/jnci/djv326.
8. Wang, P.H.; Lee, W.L.; Juang, C.M.; Yang, Y.H.; Lo, W.H.; Lai, C.R.; Hsieh, S.L.; Yuan, C.C. Altered mRNA expressions of sialyltransferases in ovarian cancers. *Gynecol. Oncol.* **2005**, *99*, 631–639, doi:10.1016/j.ygyno.2005.07.016.
9. Burchell, J.; Poulsom, R.; Hanby, A.; Whitehouse, C.; Cooper, L.; Clausen, H.; Miles, D.; Taylor-Papadimitriou, J. An alpha2,3 sialyltransferase (ST3Gal I) is elevated in primary breast carcinomas. *Glycobiology* **1999**, *9*, 1307–1311.
10. Schneider, F.; Kemmner, W.; Haensch, W.; Franke, G.; Gretschel, S.; Karsten, U.; Schlag, P.M. Overexpression of sialyltransferase CMP-sialic acid:Galbeta1,3GalNAc-R alpha6-Sialyltransferase is related to poor patient survival in human colorectal carcinomas. *Cancer Res.* **2001**, *61*, 4605–4611.
11. Kudo, T.; Ikehara, Y.; Togayachi, A.; Morozumi, K.; Watanabe, M.; Nakamura, M.; Nishihara, S.; Narimatsu, H. Up-regulation of a set of glycosyltransferase genes in human colorectal cancer. *Lab. Invest.* **1998**, *78*, 797–811.
12. Videira, P.A.; Correia, M.; Malagolini, N.; Crespo, H.J.; Ligeiro, D.; Calais, F.M.; Trindade, H.; Dall'Olio, F. ST3Gal.I sialyltransferase relevance in bladder cancer tissues and cell lines. *BMC Cancer* **2009**, *9*, 357, doi:10.1186/1471-2407-9-357.

13. Liang, Y.J.; Ding, Y.; Levery, S.B.; Lobaton, M.; Handa, K.; Hakomori, S.I. Differential expression profiles of glycosphingolipids in human breast cancer stem cells vs. cancer non-stem cells. *Proc. Natl. Acad. Sci. USA* **2013**, *110*, 4968–4973, doi:10.1073/pnas.1302825110.
14. Westrick, M.A.; Lee, W.M.; Macher, B.A. Isolation and characterization of gangliosides from chronic myelogenous leukemia cells. *Cancer Res.* **1983**, *43*, 5890–5894.
15. Merritt, W.D.; Sztein, M.B.; Taylor, B.; Reaman, G.H. Immunoreactivity of leukemic lymphoblasts of T-cell and B-cell precursor origin with monoclonal anti-GD3 and anti-GM3 antibodies. *Leukemia* **1991**, *5*, 1087–1091.
16. Sun, M.; Zhao, X.; Liang, L.; Pan, X.; Lv, H.; Zhao, Y. Sialyltransferase ST3GAL6 mediates the effect of microRNA-26a on cell growth, migration, and invasion in hepatocellular carcinoma through the protein kinase B/mammalian target of rapamycin pathway. *Cancer Sci.* **2017**, *108*, 267–276, doi:10.1111/cas.13128.
17. Glavey, S.V.; Manier, S.; Natoni, A.; Sacco, A.; Moschetta, M.; Reagan, M.R.; Murillo, L.S.; Sahin, I.; Wu, P.; Mishima, Y., et al. The sialyltransferase ST3GAL6 influences homing and survival in multiple myeloma. *Blood* **2014**, *124*, 1765–1776, doi:10.1182/blood-2014-03-560862.
18. Uhlen, M.; Fagerberg, L.; Hallstrom, B.M.; Lindskog, C.; Oksvold, P.; Mardinoglu, A.; Sivertsson, A.; Kampf, C.; Sjostedt, E.; Asplund, A., et al. Proteomics. Tissue-based map of the human proteome. *Science* **2015**, *347*, 1260419, doi:10.1126/science.1260419.
19. Uhlen, M.; Zhang, C.; Lee, S.; Sjostedt, E.; Fagerberg, L.; Bidkhori, G.; Benfeitas, R.; Arif, M.; Liu, Z.; Edfors, F., et al. A pathology atlas of the human cancer transcriptome. *Science* **2017**, *357*, doi:10.1126/science.aan2507.
20. Human Protein Atlas. Retrieved June 20, 2018, from https://www.proteinatlas.org/.
21. Reticker-Flynn, N.E.; Bhatia, S.N. Aberrant glycosylation promotes lung cancer metastasis through adhesion to galectins in the metastatic niche. *Cancer Discov.* **2015**, *5*, 168–181, doi:10.1158/2159-8290.CD-13-0760.
22. Stremitzer, S.; Berghoff, A.S.; Volz, N.B.; Zhang, W.; Yang, D.; Stintzing, S.; Ning, Y.; Sunakawa, Y.; Yamauchi, S.; Sebio, A., et al. Genetic variants associated with colorectal brain metastases susceptibility and survival. *Pharm. J.* **2017**, *17*, 29–35, doi:10.1038/tpj.2015.86.
23. Bos, P.D.; Zhang, X.H.; Nadal, C.; Shu, W.; Gomis, R.R.; Nguyen, D.X.; Minn, A.J.; van de Vijver, M.J.; Gerald, W.L.; Foekens, J.A., et al. Genes that mediate breast cancer metastasis to the brain. *Nature* **2009**, *459*, 1005–1009, doi:10.1038/nature08021.
24. Hsu, M.K.; Wu, I.C.; Cheng, C.C.; Su, J.L.; Hsieh, C.H.; Lin, Y.S.; Chen, F.C. Triple-layer dissection of the lung adenocarcinoma transcriptome: regulation at the gene, transcript, and exon levels. *Oncotarget* **2015**, *6*, 28755–28773, doi:10.18632/oncotarget.4810.
25. Senda, M.; Ito, A.; Tsuchida, A.; Hagiwara, T.; Kaneda, T.; Nakamura, Y.; Kasama, K.; Kiso, M.; Yoshikawa, K.; Katagiri, Y., et al. Identification and expression of a sialyltransferase responsible for the synthesis of disialylgalactosylgloboside in normal and malignant kidney cells: downregulation of ST6GalNAc VI in renal cancers. *Biochem. J.* **2007**, *402*, 459–470, doi:10.1042/BJ20061118.
26. Tsuchida, A.; Okajima, T.; Furukawa, K.; Ando, T.; Ishida, H.; Yoshida, A.; Nakamura, Y.; Kannagi, R.; Kiso, M.; Furukawa, K. Synthesis of disialyl Lewis a (Le(a)) structure in colon cancer cell lines by a sialyltransferase, ST6GalNAc VI, responsible for the synthesis of alpha-series gangliosides. *J. Biol. Chem.* **2003**, *278*, 22787–22794, doi:10.1074/jbc.M211034200.
